# Cultural activities and all-cause mortality among Finnish adults: a 19-year follow-up

**DOI:** 10.1186/s12889-025-23032-4

**Published:** 2025-05-23

**Authors:** Tellervo Nenonen, Anne Kouvonen, Tommi Härkänen, Tuija Martelin

**Affiliations:** 1 Faculty of Social Sciences, University of Helsinki, Unioninkatu 37, P.O. Box 54, Helsinki, 00014 Finland; 2 Public Health, Finnish Institute for Health and Welfare, Mannerheimintie 164, P.O Box 30, Helsinki, 00271 Finland; 3 Centre for Public Health, Institute of Clinical Science, Queen’s University Belfast, Royal Victoria Hospital, Block A Northern Ireland, Belfast, BT12 6BA UK

**Keywords:** Cultural activity, All-cause mortality, Longitudinal study

## Abstract

**Background:**

The association between active cultural and leisure participation and lower all-cause mortality is well established. However, less is known about the specific role of different cultural activities. Baseline data from 2000, combined with mortality data up to 2019, provide a unique opportunity to examine the association between cultural activity and all-cause mortality.

**Methods:**

We examined this association using data from the Finnish Health 2000 study (6,548 participants aged 30 years and older), linked to mortality follow-up data (1,789 deaths) up to the end of 2019 obtained from Statistics Finland. Cultural activity was measured as: 1) event participation (visiting cultural events or going to the theater, cinema, concerts, and art exhibitions); 2) reading and listening to music (reading books, listening to records and tapes); and 3) artistic and productive activities (singing, playing an instrument, painting, handicrafts, photography and collecting). Hazard ratios were estimated using the Cox proportional hazards models, adjusting for key sociodemographic variables, health, and health behaviors. Men and women were analyzed separately.

**Results:**

For women, intermediate levels of engagement in all three domains as well as high levels of engagement in event participation and artistic and productive activities were associated with a lower risk of mortality. For men, intermediate and high levels of engagement in event participation as well as high levels of engagement in artistic and productive activities were associated with a lower risk of mortality.

**Conclusions:**

The results corroborate the findings of earlier research showing that cultural activity is associated with lower all-cause mortality. However, the possibility of reversed causality or the role of unmeasured factors cannot be ruled out, even though a range of relevant confounders were controlled for. Assessing the mechanisms behind the association was beyond the scope of this study. The study suggests that engagement in cultural activities could be one factor associated with longevity, but more research is needed to examine the causality.

**Supplementary Information:**

The online version contains supplementary material available at 10.1186/s12889-025-23032-4.

## Introduction

Active engagement in arts, cultural or leisure activities as a precursor to good health, life satisfaction and reduced mortality is a topic that has been increasingly studied in recent decades [1, 2]. However, some activities may be more beneficial than others, and outcomes may vary across genders and populations. Purely cultural activities may also have different mechanisms for enhancing health and longevity than other leisure activities [3]. A recent review mapped over 600 mechanisms by which cultural and leisure-time activities can impact health [4]. These mechanisms can be categorized as psychological, biological, social, and behavioral processes that operate at individual (micro), group (meso), and societal (macro) levels. For example, engaging in cultural activities can reduce a person’s stress responses or elevate their mood, and sharing experiences or for example singing together can enhance the feeling of belonginess. Furthermore, attending and organizing cultural occasions can enrich people’s lives at a community level. The mechanisms are often studied with interventions or experiments (see for example [5–7]) and are difficult to demonstrate in population level studies. Nevertheless, research on associations between cultural activities and population level well-being and longevity is important, and together with intervention-based research, it can bring valuable knowledge for public health planners and policy makers.

In a 1978–2005 study of the Finnish adult population aged 30–59 years, active participation predicted longer survival in a 20-year follow-up for men when controlling for age, smoking, alcohol consumption, obesity, self-rated health, and diagnosed chronic diseases [8]. In a 26-year follow-up among 18- to 65-year-old Swedish adults, mortality was lower for those who engaged in leisure-time activities when controlling for age, gender, health, and alcohol consumption [9]. In another Swedish study, certain cultural activities (going to the cinema, concerts, museums or art exhibitions) were associated with a lower risk of mortality in a 14-year follow-up, whereas attending theater plays, church services or sports events as a spectator, and reading and making music were not associated with mortality [10]. In a further earlier Swedish study, frequent attendance at cultural events was associated with reduced mortality, while reading books or periodicals, making music or singing in a choir were not [11].

The association between cultural engagement and longevity has also been demonstrated to a certain extent among the older population. In older Swedish adults, gardening and engaging in hobbies were associated with a decreased risk of mortality after controlling for health variables [12]. However, in another study, the same activities were only associated with longer survival in older men, while older women seemed to benefit more from social activities [13]. In a recent study of older Japanese adults, a linear association was found between the number of leisure activities and reduced risk of mortality. However, activities that were group-based or involved physical activity were the strongest predictors [14]. A partially similar result was found in another Japanese study, in which participation in solitary physical activities and in group cultural activities reduced the risk of incident frailty in those aged 65 years or older [15]. In a study of the US population with two cohorts (2012 and 2014, followed through 2016), participants aged = 65 years had a higher mortality risk if they did not engage in listening to music, singing/playing an instrument, or doing arts and crafts. Participants aged < 65 years had a higher mortality risk if they did not listen to music. Lastly, older participants in the 2014 cohort had a higher mortality risk if they did not engage in active arts [16].

A previous study (1986–2004) in the Finnish working-age population found that high engagement in cultural activities was associated with decreased all-cause mortality, as well as with a reduced risk of cardiovascular mortality and mortality from external causes of death (such as accidents, violence, etc.). The associations with all-cause mortality and death from external causes remained after adjustment for behavioral risk factors, leading the authors to conclude that the improved overall survival of culturally engaged employees was largely attributable to their lower risk of death from external causes [17]. A recent Welsh study, adjusting for age, sex, socioeconomic position (SEP), and lifestyle behaviors, found that overall cultural participation was associated with better self-rated health (SRH), and a lower risk of cardiovascular disease and anxiety or depression. However, there was no association between local authority cultural expenditure and local life expectancy [18]. A Swedish study on urban residents showed that frequent attendance at cinemas, theaters, art galleries, live music shows, and museums was associated with a lower risk of cancer-related mortality [19]. Furthermore, a Norwegian study indicated that frequent participation in both receptive and creative cultural activities was associated with a lower risk of cardiovascular and cancer-related mortality [20]. A recent Swedish study showed that not going to the theater/cinema in the past year was associated with higher all-cause and cardiovascular mortality, while not going to an art exhibition was associated with higher all-cause and other-cause mortality. Furthermore, the combination of neither going to the theater/cinema nor to an art exhibition during the past year was associated with higher all-cause, cardiovascular and other-cause mortality [21].

Taken together, the association between cultural activity and reduced mortality is well established. However, there is a lack of recent research in the Finnish context, as prior research reaches only to 2004/2005 [8, 17] and partly only focuses on working age population. Our data provide a unique opportunity to assess the long-term association between cultural activity and mortality in the adult population. By analyzing the different activities and women and men separately, it is possible to examine the effects of more “purely” cultural activities and to take the possible gender differences into consideration. In observational studies and population level studies like this, causality is difficult to demonstrate, as cultural participation may serve as a proxy for other factors, such as social capital and factors related to socio-economic status [22, 23]. Furthermore, individual characteristics or for example mental health and cognitive challenges can be barriers for cultural engagement [24] and even be associated with the mortality outcome.

The aim of this study was to examine the association between three different kinds of cultural activities (event participation, reading and listening to music, and artistic and productive activities) and all-cause mortality in the general Finnish population aged 30 years and older from 2000 to 2019. Key socio-demographic, health, and health behavior variables that are associated with mortality were controlled for. Furthermore, in subsequent analyses the deaths occurring during the first three years of the follow-up were excluded, to partly control the possibility for reversed causality.

## Data and methods

The baseline data were derived from the Health 2000 Survey, a population-based survey of adults aged 30 years and older conducted in 2000–2001 based on two-stage stratified sampling [25]. The data were collected through interviews, questionnaires, measurements, and clinical examinations. The sample size was 8,028, 93% of whom responded to the questionnaires providing information on health and functional capacity, 88% participated in the health interview, and 85% took part in the clinical examination. The survey was approved by the Ethics Committee for Epidemiology and Public Health in the Hospital District of Helsinki and Uusimaa. All participants gave written informed consent, including consent to link survey to register data [25, 26]. The follow-up data on mortality were obtained from the Finnish National Registry for Cause of Death, which covers 100% of the population [27]. Participants were linked to the Registry for Cause of Death using their national identification numbers, and their possible mortality was followed from 2000 to 2019.

The final analytical sample consisted of 6,548 participants (with complete responses for the cultural activity variables), and the number of deaths that occurred by 2019 was 1,789 in this subset. In the analytical sample the max age was 99 years, mean age 53 years, and the standard deviation 15.5.

### Variables

Socio-demographic factors and other covariates included age, gender (man/woman), area of residence (urban/rural), marital status (married vs other), years of education (0–8, 9–12 or > 12 years) and financial difficulties (no = enough money for family needs, yes = need to negotiate costs at least to some extent). Health and health behavioral variables included obesity (measured by weight and height in kg/m^2^, with obesity defined using the cut-off point of 30 kg/m^2^), presence of chronic illness, smoking, alcohol consumption (calculated as quantity of absolute alcohol per week, divided into equivalents of harmful consumption: 14 or more servings/week (168 g or more) for men, 7 or more servings/week (84 g or more) for women), good self-rated health (good or fairly good = good; moderate, rather poor, or poor = poor) [28], common mental disorder (General Health Questionnaire (GHQ) score > 3 indicating mental disorder) [29], and physical activity (leisure time spent doing at least 4 h of walking, cycling or other physical activity per week indicating physical activity, leisure time spent reading, watching TV and other non-physical activities indicating no physical activity).

Engagement in cultural activities was measured with three questions, each covering one activity domain: 1) How often do you go to the theater, cinema, concerts, art exhibitions, sports events, and so on? (event participation); 2) How often do you read literature, listen to records and tapes? (reading and listening to music); and 3) How often do you engage in handicrafts or hobby crafts, play an instrument, sing, engage in photography, painting, collecting, and so on? (artistic and productive activities). The response options for each question were: “Every day or most days = 5”, “Once or twice a week = 4”, “Once or twice a month = 3”, “Once or a few times a year = 2”, and “Rarely or never = 1”.

Each activity domain was analyzed separately by dividing the population into quartiles as precisely as possible, identifying three engagement groups: low levels of engagement in activity (25%), intermediate levels of engagement in activity (50%) and high levels of engagement in activity (25%). In the case of event participation, at least once or twice a month was classified as high levels of engagement (30%), once or a few times a year as intermediate levels of engagement (53%), and rarely or never as low levels of engagement (19%). In the case of reading and listening to music, every day or most days was classified as high levels of engagement (33%), once or twice a week and once or twice a month as intermediate levels of engagement (44%), and once or a few times a year, and rarely or never, as low levels of engagement (23%). Lastly, in the case of artistic and productive activities, every day or most days and once or twice a week were classified as high levels of engagement (33%), once or twice a month and once or a few times a year as intermediate levels of engagement (39%), and rarely or never as low levels of engagement (28%). The categories were collapsed into quartiles and halves to follow earlier Finnish studies [8, 17] and other studies in this field [20], as well as to make the different activity domains comparable to each other in this study. It is for example more usual to engage regularly (perhaps even daily) with reading and listening to music or with handicrafts and playing an instrument, than to go to events like theater or concerts.

### Analysis

All analyses were performed using SPSS 29 complex samples procedures to account for the sampling design and post-stratification weights. Mortality was analyzed using the Cox proportional hazards models, separately for women and men. The analyses were first adjusted for age, and in the second model additionally for all other control variables. In the third model, deaths occurring during the first three years of follow-up were censored and the analyses were adjusted for the same factors as in the second model. This was done to exclude the deaths that would have occurred soon regardless of the engagement level, following the procedures in earlier studies [8, 20] and also to reduce the possibility of reverse causality. Hazard ratios (HRs) with their 95% confidence intervals (CIs) were reported for all models.

## Results

Data for 2,923 (45%) men and 3,625 (55%) women were included in the analyses (Table 1). The mean age was slightly higher in women (54 years, standard error 0.24) than in men (51 years, standard error 0.28), as was the proportion of those with a high level of education and chronic illness. Common mental disorders (GHQ score over 3) were more common in women than in men, while smoking and high alcohol consumption were more common in men.

**Table 1:** Key characteristics of the participants (unweighted counts; weighted means and prevalences)

Characteristic	All	Women	Men
n	6548	3625	2923
Number of deaths	1789	972	817
Mean time of follow-up	16	16	16
Mean age (years)	52	54	51
Rural residence (%)	24	23	25
Married (%)	20	21	20
High education (%)	28	32	24
Financial difficulties (%)	40	41	39
Obesity (%)	22	24	21
Chronic illness (%)	52	54	49
Smoking (%)	27	22	34
High alcohol consumption (%)	16	11	21
Good self-rated health (%)	62	63	62
Common mental disorder (%)	19	21	17
Physical activity (4 h per week or more) (%)	72	72	72
High levels of engagement: Going to the theater, cinema, concerts, etc. (%)	23	21	25
High levels of engagement: Reading, listening to music (%)	32	40	24
High levels of engagement: Handicrafts, singing, painting, etc. (%)	33	43	21

HRs for cultural activity variables are shown in Tables 2 and 3, and HRs for control variables in the Appendix (Tables 1–6). Among women, for event participation and artistic and productive activities, there were associations between both high and intermediate levels of engagement in activity and a lower risk of death in model 3 (Table 2). HRs for mortality were approximately similar in the groups with intermediate and high levels of engagement. For example, regarding event participation, in model 3 the HR for mortality for women with high levels of engagement was 0.73 (CI 0.55–0.96), and the corresponding HR for women with intermediate levels of engagement was 0.71 (0.60–0.85). For reading and listening to music, there were associations between intermediate levels of engagement in activity and a lower risk of mortality in models 2 and 3. In model 3, the HR for women with intermediate levels of engagement in reading and listening to music was 0.77 (CI 0.62–0.95) compared with women with low levels of engagement. In addition, when controlling for any of the cultural activities and all (other) confounders, being older, non-married, smoker, and having suboptimal self-rated health and not engaging in physical activity were associated with a higher risk of death in women (Supplementary Material: Tables 1, 3 and 5).

**Table 2:** Hazard ratios and their 95% confidence intervals for women: risk of death compared with individuals with low (lowest quartile) levels of engagement in activity (Intermediate = two middle quartiles, high = highest quartile), unweighted counts (women n=3625)

Women Activity domain	Model 1 Engagement levels:		Model 2 Engagement levels:		Model 3 Engagement levels:	
	Intermediate	High^a^	Intermediate	High^b^	Intermediate	High^c^
Event participation	0.61*** (0.53, 0.70)	0.55*** (0.43, 0.70)	0.74*** (0.63, 0.88)	0.76 (0.58, 1.00)	0.71*** (0.60, 0.85)	0.73* (0.55, 0.96)
Reading, listening to music	0.72*** (0.60, 0.84)	0.84* (0.72, 0.96)	0.77* (0.62, 0.94)	0.97 (0.80, 1.17)	0.77* (0.62, 0.95)	0.98 (0.80, 1.19)
Artistic and productive activities	0.58*** (0.49, 0.70)	0.56*** (0.49, 0.65)	0.74** (0.61, 0.90)	0.73** (0.61, 0.88)	0.75* (0.61, 0.92)	0.71*** (0.59, 0.85)

19-year follow-up: Model 1 = adjusted for age; Model 2 = adjusted for age, area of residence, marital status, education, financial difficulties, obesity, chronic illness, smoking, alcohol consumption, self-rated health, common mental disorder and physical activity; Model 3 = adjusted for age, area of residence, marital status, education, financial difficulties, obesity, chronic illness, smoking, alcohol consumption, self-rated health, common mental disorder and physical activity; censored for deaths occurring in first 3 years of follow-up. Note: *p < 0.05, **p < 0.01, ***p < 0.001

^a^Event participation, high levels of engagement in activity=at least once or twice a month

^b^Reading and listening to music, high levels of engagement in activity=every day or most days

^c^Artistic and productive activities, high levels of engagement in activity= every day or most days and once or twice a week

**Table 3:** Hazard ratios and their 95% confidence intervals for men: risk of death compared with individuals with low (lowest quartile) levels of engagement in activity (Intermediate = two middle quartiles, high = highest quartile), unweighted counts (men n=2923)

Men Activity domain	Model 1 Engagement levels:		Model 2 Engagement levels:		Model 3 Engagement levels:	
	Intermediate	High^a^	Intermediate	High^b^	Intermediate	High^c^
Event participation	0.57*** (0.50, 0.66)	0.50*** (0.40, 0.63)	0.72*** (0.61, 0.84)	0,70** (0.55, 0.89)	0.75** (0.64, 0.89)	0.68** (0.52, 0.87)
Reading and listening to music	0.80*** (0.70, 0.92)	0.84** (0.71, 1.00)	0.98 (0.83, 1.16)	0.92 (0.75, 1.12)	0.97 (0.81, 1.16)	0.90 (0.73, 1.10)
Artistic and productive activities	0.74*** (0.65, 0.85)	0.61*** (0.50, 0.74)	0.91 (0.78, 1.06)	0.80** (0.65, 1.00)	0.92 (0.78, 1.08)	0.81** (0.66, 1.00)

19-year follow-up: Model 1 = adjusted for age; Model 2 = adjusted for age, area of residence, marital status, education, financial difficulties, obesity, chronic illness, smoking, alcohol consumption, self-rated health, common mental disorder and physical activity; Model 3 = adjusted for age, area of residence, marital status, education, financial difficulties, obesity, chronic illness, smoking, alcohol consumption, self-rated health, common mental disorder and physical activity; censored for deaths occurring in first 3 years of follow-up. Note: *p < 0.05, **p < 0.01, ***p < 0.001

^a^Event participation, high levels of engagement in activity=at least once or twice a month

^b^Reading and listening to music, high levels of engagement in activity=every day or most days

^c^Artistic and productive activities, high levels of engagement in activity= every day or most days and once or twice a week

In a similar way high and intermediate levels of engagement in event participation were associated with a lower risk of death among men (Table 3). In model 3, men with high levels of engagement had a HR of 0.68 (CI 0.52–0.87) compared with men with low levels of engagement, and men with intermediate levels of engagement had a HR of 0.75 (CI 0.64–0.89). For artistic and productive activities, there were statistically significant associations in models 2 and 3 for high levels of engagement. In model 3, men with high levels of engagement had a HR of 0.81 (CI 0.66–1.00) compared with men with low levels of engagement. Furthermore, when controlling for any of the cultural activities and all (other) confounders, being older, non-married, a smoker, a risky alcohol drinker and having suboptimal self-rated health and having a chronic illness were associated with a higher risk of death among men. Regarding reading and listening to music, also low education was significantly associated with a higher risk of death in men (Supplementary Material: Tables 2, 4 and 6).

## Discussion

The present study examined the association between three domains of cultural activity in 2000 and all-cause mortality until 2019 in the general Finnish population aged 30 years and older. For women, intermediate levels of engagement in all three domains as well as high levels of engagement in event participation and artistic and productive activities were associated with a lower risk of mortality. For men, intermediate and high levels of engagement in event participation as well as high levels of engagement in artistic and productive activities were associated with a lower risk of mortality.

In general, the results are consistent with prior research on the association between cultural activities and longevity. There may be several possible mechanisms behind the association. For example, reading books has been shown to increase longevity in older adults, and the survival advantage may work through a cognitive mediator [30]. Furthermore, research has shown that music may have a stress-reducing effect on the neurological and immunological systems of individuals. Music, singing and theater engagement have been shown to have therapeutic effects on cancer patients in clinical studies [20, 31–33]. Choir singing has been associated with greater health satisfaction and quality of life among the elderly as well as with better emotional and social functioning and health among disadvantaged adults [34, 35].

Even though there is large evidence about different mechanisms by which cultural activities can enhance health and longevity, in our study it is not possible to rule out the possibility of underlying confounding factors such as for example low cognitive ability or social exclusion. Cultural activity chronologically precedes mortality, but there could be reversed causality in terms of selection into better health and thus to more resources for leading a culturally active lifestyle. However, our models were adjusted for age, marital status, education, long-term illness, health behaviors, and common mental disorder. Not being married was associated with a higher risk for mortality for both women and men, and having a long-term illness was associated with a higher risk for mortality for men. There were also significant associations between health behavioral factors and mortality. Furthermore, self-rated health was associated with mortality in all cases but having a common mental disorder was not.

The associations between cultural activity and mortality were in most cases mitigated, but not abolished, after adding the covariates into the models. Regarding reading and listening to music and intermediate levels of engagement in artistic and productive activities among men, and regarding high levels of engagement in reading and listening to music among women, the associations with mortality only persisted in the model with only age as a covariate. The possibility of reversed causality (for example having an illness that would lead to death despite of the cultural activity and perhaps hinder the participation) is also partly controlled for by excluding the deaths that occurred during the first three years of the follow-up.

Precise comparisons with earlier studies are somewhat difficult because of differences in operationalizing cultural activities [20]. In a Swedish 14-year follow-up, going to the cinema, concerts, museums or art exhibitions was associated with a lower risk of mortality, while attending theater plays, church services, or sports events as a spectator, reading and making music were not [9]. In our study, going to the theater, cinema, concerts, art exhibitions, and sports events were associated with a lower risk of death for both women and men. Similarly, in an earlier Swedish study, reading, making music and singing in a choir were not predictors of longer survival [10], but in our study they were for women. However, our variable of making music and singing also covered handicrafts, painting, collecting, and so on.

Our results mirror the findings in earlier Finnish studies to some extent. In the 2005 Finnish study, active participation predicted longer survival for middle-aged men, but not for women [8]. In our study, women benefited from all three cultural activity categories, while men mainly benefited from event participation. However, the activity variable in the earlier Finnish study also covered involvement in clubs and voluntary societies, religious engagement, outdoor and productive activity, and studying. Perhaps our variable is more applicable to women than to men. The item ‘spectator at sports events’, which was included in event participation, might be more applicable to men, while, for example, the item ‘handicrafts’ in artistic and productive activities could be more applicable to women. A previous study of the Finnish working-age population in 2004 found that high engagement in cultural activities was associated with decreased all-cause mortality as well as with a reduced risk of cardiovascular mortality and mortality from external causes of death [17]. In that study, the activity item also included activities in associations, studying, and social activities.

In a study of the US population participants aged < 65 years had a higher mortality risk if they did not listen to music. This result is consistent with our finding in women, even though the follow-up time in the US study was much shorter [16]. Lastly, a Swedish study showed that not going to the theater/cinema in the past year was associated with higher all-cause and cardiovascular mortality, while not visiting an art exhibition was associated with higher all-cause and other-cause mortality. Furthermore, the combination of neither going to the theater/cinema nor to an art exhibition in the past year was associated with higher all-cause, cardiovascular and other-cause mortality [21]. Our study only assessed all-cause mortality, but the results are consistent with our findings for both women and men.

Our finding that cultural activities were more distinctly associated with lower mortality among women than among men could stem from the measurement being more applicable to women. Alternatively, it could indicate that as women may be more responsible for caregiving tasks, it might be more beneficial for them to get opportunities for cultural and recreational activities. On the other hand, as the life expectancy in Finland for men is lower than for women, it could be even more important to enhance the longevity in men, and the knowledge about the associations between cultural activities and mortality could be one aspect to utilize in that. However, more research that could help ascertain the direction of causality in the population level, possibly by utilizing instrumental variables or triangulation, would be needed.

### Strengths and limitations

The strengths of this study are the representative, high-quality survey data linked to the death registry, which enabled us to examine the association between cultural activity and all-cause mortality, while controlling for relevant confounders. A particular further strength is the long follow-up time of 19 years. The response rates of the different parts of the study were very high (85% to 93%) and the data are representative of the entire Finnish adult (aged 30 +) population [26].

Our cultural activity variables covered a wide range of activities, and the operationalization is therefore comprehensive. One limitation, however, is that each activity domain (one item in the questionnaire) covers somewhat different types of activities, and the event participation domain even includes being a spectator at sports events. Hence, it is not possible to clearly distinguish the role of different activities. The item ‘spectator at sports events’ might be more applicable to men, while, for example, the ‘handicrafts’ item could be more applicable to women, although in an earlier study, some male participants considered repairing old cars to be a handicraft [36]. On the other hand, the artistic and productive activities domain covers a wide range of activities that could be performed by men (photography, singing, playing an instrument, collecting, etc.) Furthermore, in this study, we were not able to look at the tenure of the activities, which has been shown to be a strong predictor of good self-reported health and could thus play a role in longevity as well [37].

Lastly, in this study we were not able to control for all possible confounders such as for example cognitive ability. Furthermore, as this was an observational study, it is not possible to demonstrate causality. However, we controlled for key socio-demographic, health and health behavior related variables, and in the last models we excluded the deaths occurring during the first three years of the follow-up.

## Conclusions

Our 19-year follow-up study showed that for women, intermediate levels of engagement in all three domains as well as high levels of engagement in event participation and artistic and productive activities were associated with a lower risk of mortality. For men, intermediate and high levels of engagement in event participation as well as high engagement in artistic and productive activities were associated with a lower risk of mortality. While some of the items in our cultural activity variable may be more applicable to women, the results emphasize the importance of cultural and recreational activity in the context of longevity particularly in women. As women may be more responsible for caregiving activities, providing them with opportunities for cultural participation could enhance their well-being and ultimately longevity. However, encouraging more active participation in various events among men might be one of the measures to narrow the gap in life expectancy between men and women as the life expectancy for women is higher than for men in Finland [38]. More research to confirm the causality is needed, but together with the prior research about the mechanisms of cultural activities as predecessors of longevity, the results could be utilized in population health promotion.

## Supplementary Information

Supplementary Material 1.

## Data Availability

In accordance with the data protection regulations of the national register-holders, we cannot make the data directly available to third parties. Interested researchers may contact the register-holding public institution Statistics Finland (http://www.stat.fi/tup/mikroaineistot/index_en.html) for register data and the Finnish Institution for Health and Welfare for health survey data.
