# Supplementary material for: Cultural activities and all-cause mortality among Finnish adults: a 19-year follow-up
Source: BMC Public Health. 2025 May 23;25:1906. doi: 10.1186/s12889-025-23032-4 (PMC12100814; doi:10.1186/s12889-025-23032-4)
Supplement: Supplementary file 1 — Supplementary Material 1. [file 12889_2025_23032_MOESM1_ESM.docx]

| Predictor |  | Model 1 |  |  |  | Model 2 | |  |  | Model 3 | |
| --- | --- | --- | --- | --- | --- | --- | --- | --- | --- | --- | --- |
|  | HR | Lower | Upper |  | HR | Lower | Upper |  | HR | Lower | Upper |
| **Age (+1 year)** | 1.12*** | 1.12 | 1.13 |  | 1.12*** | 1.11 | 1.13 |  | 1.12*** | 1.11 | 1.13 |
| **Area of residence (urban vs. rural)** |  |  |  |  | 0.93 | 0.81 | 1.07 |  | 0.96 | 0.83 | 1.11 |
| **Marital status (non-married vs. married/cohabiting)** |  |  |  |  | 1.41*** | 1.20 | 1.67 |  | 1.46*** | 1.23 | 1.73 |
| **Education** |  |  |  |  |  |  |  |  |  |  |  |
| 0-8 years vs. > 12 years |  |  |  |  | 0.99 | 0.79 | 1.24 |  | 0.96 | 0.76 | 1.21 |
| 9-12 years vs. > 12 years |  |  |  |  | 1.05 | 0.83 | 1.32 |  | 1.04 | 0.82 | 1.32 |
| **Financial difficulties no vs. yes** |  |  |  |  | 0.99 | 0.85 | 1.15 |  | 0.97 | 0.83 | 1.14 |
| **Obesity no vs. yes** |  |  |  |  | 1.03 | 0.87 | 1.21 |  | 1.03 | 0.86 | 1.22 |
| **Chronic illness (no vs. yes)** |  |  |  |  | 0.83 | 0.68 | 1.00 |  | 0.85 | 0.70 | 1.04 |
| **Smoking no vs. yes** |  |  |  |  | 0.58 | 0.45 | 0.74 |  | 0.59*** | 0.46 | 0.76 |
| **Self-rated health (poor/fairly poor/intermediate vs. fairly good/good)** |  |  |  |  | 1.39*** | 1.18 | 1.63 |  | 1.40*** | 1.19 | 1.64 |
| **Physical activity no vs. yes** |  |  |  |  | 1.40*** | 1.21 | 1.62 |  | 1.37*** | 1.18 | 1.59 |
| **Alcohol use no vs. yes** |  |  |  |  | 1.01*** | 0.72 | 1.41 |  | 0.92 | 0.66 | 1.30 |
| **Common mental disorder no vs. yes** |  |  |  |  | 0.93 | 0.77 | 1.13 |  | 0.99 | 0.81 | 1.21 |
| **Levels of engagement in cultural activity** |  |  |  |  |  |  |  |  |  |  |  |
| high vs. low | 0.55*** | 0.43 | 0.70 |  | 0.76 | 0.58 | 1.00 |  | 0.73* | 0.55 | 0.96 |
| intermediate vs. low | 0.61*** | 0.53 | 0.70 |  | 0.74*** | 0.63 | 0.88 |  | 0.71*** | 0.60 | 0.85 |

Appendix table 1. Hazard ratios and 95 % confidence intervals for women. Cultural activity: Event participation.

19-year follow-up: Model 1=adjusted for age; Model 2=adjusted for age, area of residence, marital status, education, financial difficulties, obesity, chronic illness, smoking, alcohol consumption, self-rated health, common mental disorder and physical activity; Model 3=adjusted for age, area of residence, marital status, education, financial difficulties, obesity, chronic illness, smoking, alcohol consumption, self-rated health, common mental disorder and physical activity, censored for deaths occurring in first 3 years of follow-up. Note: *p<0.05, **p<0.01, ***p<0.001

Appendix table 2. Hazard ratios and 95 % confidence intervals for men. Cultural activity: Event participation.

|  |  | Model 1 |  |  |  | Model 2 |  |  |  | Model 3 |  |
| --- | --- | --- | --- | --- | --- | --- | --- | --- | --- | --- | --- |
| Predictor | HR | Lower | Upper |  | HR | Lower | Upper |  | HR | Lower | Upper |
| **Age (+1 year)** | 1.09*** | 1.09 | 1.10 |  | 1.10*** | 1.09 | 1.11 |  | 1.10*** | 1.09 | 1.11 |
| **Area of residence (urban vs. rural)** |  |  |  |  | 1.03 | 0.85 | 1.25 |  | 1.03 | 0.85 | 1.24 |
| **Marital status (non-married vs. married/cohabiting)** |  |  |  |  | 1.36*** | 1.16 | 1.59 |  | 1.40*** | 1.19 | 1.64 |
| **Education** |  |  |  |  |  |  |  |  |  |  |  |
| 0-8 years vs. > 12 years |  |  |  |  | 1.17 | 0.96 | 1.43 |  | 1.17 | 0.96 | 1.44 |
| 9-12 years vs. > 12 years |  |  |  |  | 1.00 | 0.79 | 1.27 |  | 0.98 | 0.77 | 1.26 |
| **Financial difficulties no vs. yes** |  |  |  |  | 0.95 | 0.83 | 1.09 |  | 0.92 | 0.80 | 1.05 |
| **Obesity no vs. yes** |  |  |  |  | 1.10 | 0.90 | 1.33 |  | 1.10 | 0.90 | 1.33 |
| **Chronic illness (no vs. yes)** |  |  |  |  | 0.72*** | 0.60 | 0.85 |  | 0.74** | 0.62 | 0.88 |
| **Smoking no vs. yes** |  |  |  |  | 0.52*** | 0.43 | 0.63 |  | 0.52*** | 0.43 | 0.64 |
| **Self-rated health poor/fairly poor/intermediate vs. fairly good/good** |  |  |  |  | 1.34*** | 1.13 | 1.59 |  | 1.33** | 1.11 | 1.59 |
| **Physical activity no vs. yes** |  |  |  |  | 1.06 | 0.88 | 1.29 |  | 1.03 | 0.85 | 1.25 |
| **Alcohol use no vs. yes** |  |  |  |  | 0.70*** | 0.57 | 0.87 |  | 0.72** | 0.57 | 0.90 |
| **Common mental disorder no vs. yes** |  |  |  |  | 0.84 | 0.69 | 1.04 |  | 0.86 | 0.70 | 1.07 |
| **Levels of engagement in cultural activity** |  |  |  |  |  |  |  |  |  |  |  |
| high vs. low | 0.50*** | 0.40 | 0.63 |  | 0.70** | 0.55 | 0.89 |  | 0.68** | 0.52 | 0.87 |
| intermediate vs. low | 0.57*** | 0.50 | 0.66 |  | 0.72*** | 0.61 | 0.84 |  | 0.75** | 0.64 | 0.89 |

19-year follow-up: Model 1=adjusted for age; Model 2=adjusted for age, area of residence, marital status, education, financial difficulties, obesity, chronic illness, smoking, alcohol consumption, self-rated health, common mental disorder and physical activity; Model 3=adjusted for age, area of residence, marital status, education, financial difficulties, obesity, chronic illness, smoking, alcohol consumption, self-rated health, common mental disorder and physical activity, censored for deaths occurring in first 3 years of follow-up. Note: *p<0.05, **p<0.01, ***p<0.001

Appendix table 3. Hazard ratios and 95 % confidence intervals for women. Cultural activity: Reading and listening to music.

| Predictor |  | Model 1 |  |  |  | Model 2 | |  |  | Model 3 | |
| --- | --- | --- | --- | --- | --- | --- | --- | --- | --- | --- | --- |
|  | HR | Lower | Upper |  | HR | Lower | Upper |  | HR | Lower | Upper |
| **Age (+1 year)** | 1.13*** | 1.12 | 1.14 |  | 1.12*** | 1.11 | 1.13 |  | 1.12*** | 1.11 | 1.13 |
| **Area of residence (urban vs. rural)** |  |  |  |  | 0.90 | 0.78 | 1.03 |  | 0.90 | 0.78 | 1.04 |
| **Marital status (non-married vs. married/cohabiting)** |  |  |  |  | 1.40*** | 1.18 | 1.65 |  | 1.43*** | 1.20 | 1.70 |
| **Education** |  |  |  |  |  |  |  |  |  |  |  |
| 0-8 years vs. > 12 years |  |  |  |  | 1.03 | 0.83 | 1.28 |  | 1.01 | 0.81 | 1.27 |
| 9-12 years vs. > 12 years |  |  |  |  | 1.07 | 0.85 | 1.34 |  | 1.05 | 0.83 | 1.34 |
| **Financial difficulties no vs. yes** |  |  |  |  | 0.96 | 0.83 | 1.12 |  | 0.95 | 0.82 | 1.11 |
| **Obesity no vs. yes** |  |  |  |  | 1.03 | 0.87 | 1.22 |  | 1.05 | 0.88 | 1.26 |
| **Chronic illness (no vs. yes)** |  |  |  |  | 0.83 | 0.68 | 1.01 |  | 0.87 | 0.72 | 1.06 |
| **Smoking no vs. yes** |  |  |  |  | 0.58*** | 0.46 | 0.75 |  | 0.60*** | 0.46 | 0.77 |
| **Self-rated health poor/fairly poor/intermediate vs. fairly good/good** |  |  |  |  | 1.44*** | 1.22 | 1.70 |  | 1.46*** | 1.25 | 1.71 |
| **Physical activity no vs. yes** |  |  |  |  | 1.45*** | 1.26 | 1.68 |  | 1.43*** | 1.22 | 1.67 |
| **Alcohol use no vs. yes** |  |  |  |  | 1.04 | 0.74 | 1.46 |  | 0.96 | 0.68 | 1.35 |
| **Common mental disorder no vs. yes** |  |  |  |  | 0.93 | 0.77 | 1.12 |  | 1.00 | 0.82 | 1.21 |
| **Levels of engagement in cultural activity** |  |  |  |  |  |  |  |  |  |  |  |
| high vs. low | 0.84* | 0.72 | 0.96 |  | 0.97 | 0.80 | 1.17 |  | 0.98 | 0.80 | 1.19 |
| intermediate vs. low | 0.72*** | 0.60 | 0.84 |  | 0.77* | 0.62 | 0.94 |  | 0.77* | 0.62 | 0.95 |

19-year follow-up: Model 1=adjusted for age; Model 2=adjusted for age, area of residence, marital status, education, financial difficulties, obesity, chronic illness, smoking, alcohol consumption, self-rated health, common mental disorder and physical activity; Model 3=adjusted for age, area of residence, marital status, education, financial difficulties, obesity, chronic illness, smoking, alcohol consumption, self-rated health, common mental disorder and physical activity, censored for deaths occurring in first 3 years of follow-up. Note: *p<0.05, **p<0.01, ***p<0.001

| Predictor |  | Model 1 |  |  | Model 2 | | | |  | | Model 3 | | |  |
| --- | --- | --- | --- | --- | --- | --- | --- | --- | --- | --- | --- | --- | --- | --- |
|  | HR | Lower | Upper |  | HR | Lower | Upper |  | | HR | Lower | Upper |  |  |
| **Age (+1 year)** | 1.10*** | 1.09 | 1.11 |  | 1.10*** | 1.09 | 1.11 |  | | 1.10*** | 1.10 | 1.11 |  |  |
| **Area of residence (urban vs. rural)** |  |  |  |  | 1.01 | 0.83 | 1.22 |  | | 0.99 | 0.82 | 1.19 |  |  |
| **Marital status (non-married vs. married/cohabiting)** |  |  |  |  | 1.39*** | 1.19 | 1.62 |  | | 1.43*** | 1.22 | 1.68 |  |  |
| **Education** |  |  |  |  |  |  |  |  | |  |  |  |  |  |
| 0-8 years vs. > 12 years |  |  |  |  | 1.24** | 1.02 | 1.51 |  | | 1.23** | 1.01 | 1.50 |  |  |
| 9-12 years vs. > 12 years |  |  |  |  | 1.02 | 0.80 | 1.29 |  | | 1.00 | 0.79 | 1.28 |  |  |
| **Financial difficulties no vs. yes** |  |  |  |  | 0.92 | 0.81 | 1.05 |  | | 0.89 | 0.78 | 1.02 |  |  |
| **Obesity no vs. yes** |  |  |  |  | 1.09 | 0.89 | 1.32 |  | | 1.09 | 0.90 | 1.32 |  |  |
| **Chronic illness (no vs. yes)** |  |  |  |  | 0.70*** | 0.59 | 0.83 |  | | 0.71*** | 0.60 | 0.86 |  |  |
| **Smoking no vs. yes** |  |  |  |  | 0.50*** | 0.41 | 0.60 |  | | 0.50*** | 0.42 | 0.60 |  |  |
| **Self-rated health poor/fairly poor/intermediate vs. fairly good/good** |  |  |  |  | 1.36*** | 1.14 | 1.62 |  | | 1.33*** | 1.11 | 1.60 |  |  |
| **Physical activity no vs. yes** |  |  |  |  | 1.10 | 0.92 | 1.33 |  | | 1.08 | 0.89 | 1.30 |  |  |
| **Alcohol use no vs. yes** |  |  |  |  | 0.71*** | 0.57 | 0.88 |  | | 0.72** | 0.58 | 0.91 |  |  |
| **Common mental disorder no vs. yes** |  |  |  |  | 0.83 | 0.68 | 1.01 |  | | 0.84 | 0.69 | 1.04 |  |  |
| **Levels of engagement in cultural activity** |  |  |  |  |  |  |  |  | |  |  |  |  |  |
| high vs. low | 0.84** | 0.71 | 1.00 |  | 0.92 | 0.75 | 1.12 |  | | 0.90 | 0.73 | 1.10 |  |  |
| intermediate vs. low | 0.80*** | 0.70 | 0.92 |  | 0.98 | 0.83 | 1.16 |  | | 0.97 | 0.81 | 1.16 |  |  |

Appendix table 4. Hazard ratios and 95 % confidence intervals for men. Cultural activity: Reading and listening to music.

19-year follow-up: Model 1=adjusted for age; Model 2=adjusted for age, area of residence, marital status, education, financial difficulties, obesity, chronic illness, smoking, alcohol consumption, self-rated health, common mental disorder and physical activity; Model 3=adjusted for age, area of residence, marital status, education, financial difficulties, obesity, chronic illness, smoking, alcohol consumption, self-rated health, common mental disorder and physical activity, censored for deaths occurring in first 3 years of follow-up. Note: *p<0.05, **p<0.01, ***p<0.001

Appendix table 5. Hazard ratios and 95 % confidence intervals for women. Cultural activity: Artistic and productive activities.

| Predictor |  | Model 1 |  |  |  | Model 2 | |  |  | Model 3 | |
| --- | --- | --- | --- | --- | --- | --- | --- | --- | --- | --- | --- |
|  | HR | Lower | Upper |  | HR | Lower | Upper |  | HR | Lower | Upper |
| **Age (+1 year)** | 1.13*** | 1.12 | 1.13 |  | 1.12*** | 1.11 | 1.13 |  | 1.12*** | 1.11 | 1.13 |
| **Area of residence (urban vs. rural)** |  |  |  |  | 0.87 | 0.75 | 1.00 |  | 0.86 | 0.74 | 1.00 |
| **Marital status (non-married vs. married/cohabiting)** |  |  |  |  | 1.40*** | 1.18 | 1.65 |  | 1.43*** | 1.20 | 1.70 |
| **Education** |  |  |  |  |  |  |  |  |  |  |  |
| 0-8 years vs. > 12 years |  |  |  |  | 1.06 | 0.86 | 1.32 |  | 1.05 | 0.84 | 1.30 |
| 9-12 years vs. >12 years |  |  |  |  | 1.09 | 0.87 | 1.36 |  | 1.07 | 0.84 | 1.36 |
| **Financial difficulties no vs. yes** |  |  |  |  | 0.98 | 0.84 | 1.13 |  | 0.98 | 0.84 | 1.14 |
| **Obesity no vs. yes** |  |  |  |  | 1.03 | 0.87 | 1.22 |  | 1.04 | 0.87 | 1.24 |
| **Chronic illness (no vs. yes)** |  |  |  |  | 0.82 | 0.68 | 1.00 |  | 0.85 | 0.70 | 1.04 |
| **Smoking no vs. yes** |  |  |  |  | 0.59*** | 0.46 | 0.75 |  | 0.59*** | 0.46 | 0.77 |
| **Self-rated health poor/fairly poor/intermediate vs. fairly good/good** |  |  |  |  | 1.39*** | 1.19 | 1.63 |  | 1.42*** | 1.22 | 1.67 |
| **Physical activity no vs. yes** |  |  |  |  | 1.40*** | 1.21 | 1.61 |  | 1.37*** | 1.18 | 1.60 |
| **Alcohol use no vs. yes** |  |  |  |  | 1.08 | 0.77 | 1.53 |  | 1.03 | 0.72 | 1.44 |
| **Common mental disorder no vs. yes** |  |  |  |  | 0.96 | 0.79 | 1.16 |  | 1.02 | 0.84 | 1.25 |
| **Levels of engagement in cultural activity** |  |  |  |  |  |  |  |  |  |  |  |
| high vs. low | 0.56*** | 0.49 | 0.65 |  | 0.73** | 0.61 | 0.88 |  | 0.71*** | 0.59 | 0.85 |
| intermediate vs. low | 0.58*** | 0.49 | 0.70 |  | 0.74** | 0.61 | 0.90 |  | 0.75* | 0.61 | 0.92 |

19-year follow-up: Model 1=adjusted for age; Model 2=adjusted for age, area of residence, marital status, education, financial difficulties, obesity, chronic illness, smoking, alcohol consumption, self-rated health, common mental disorder and physical activity; Model 3=adjusted for age, area of residence, marital status, education, financial difficulties, obesity, chronic illness, smoking, alcohol consumption, self-rated health, common mental disorder and physical activity, censored for deaths occurring in first 3 years of follow-up. Note: *p<0.05, **p<0.01, ***p<0.001

Appendix table 6. Hazard ratios and 95 % confidence intervals for men. Cultural activity: Artistic and productive activities.

| Predictor |  | | Model 1 | |  | |  | |  | | Model 2 | |  | | Model 3 | | | |  | | |  | | |
| --- | --- | --- | --- | --- | --- | --- | --- | --- | --- | --- | --- | --- | --- | --- | --- | --- | --- | --- | --- | --- | --- | --- | --- | --- |
|  | HR | Lower | | Upper | |  | | HR | | Lower | | Upper | |  | | HR | | Lower | | | Upper | | |  |
| **Age (+1 year)** | 1.10*** | 1.09 | | 1.11 | |  | | 1.10*** | | 1.09 | | 1.11 | |  | | 1.10*** | 1.10 | | | 1.11 | | |  |  |
| **Area of residence (urban vs. rural)** |  |  | |  | |  | | 1.00 | | 0.83 | | 1.21 | |  | | 0.99 | 0.83 | | | 1.19 | | |  |  |
| **Marital status (non-married vs. married/cohabiting)** |  |  | |  | |  | | 1.36*** | | 1.16 | | 1.59 | |  | | 1.41*** | 1.20 | | | 1.65 | | |  |  |
| **Education** |  |  | |  | |  | |  | |  | |  | |  | |  |  | | |  | | |  |  |
| 0-8 years vs. > 12 years |  |  | |  | |  | | 1.22** | | 1.01 | | 1.49 | |  | | 1.22 | 1.00 | | | 1.50 | | |  |  |
| 9-12 years vs. >12 years |  |  | |  | |  | | 1.01 | | 0.80 | | 1.29 | |  | | 1.00 | 0.78 | | | 1.27 | | |  |  |
| **Financial difficulties no vs. yes** |  |  | |  | |  | | 0.91 | | 0.80 | | 1.04 | |  | | 0.89 | 0.78 | | | 1.03 | | |  |  |
| **Obesity no vs. yes** |  |  | |  | |  | | 1.09 | | 0.90 | | 1.32 | |  | | 1.08 | 0.89 | | | 1.30 | | |  |  |
| **Chronic illness (no vs. yes)** |  |  | |  | |  | | 0.70*** | | 0.59 | | 0.83 | |  | | 0.71*** | 0.59 | | | 0.85 | | |  |  |
| **Smoking no vs. yes** |  |  | |  | |  | | 0.51*** | | 0.42 | | 0.63 | |  | | 0.51*** | 0.42 | | | 0.62 | | |  |  |
| **Self-rated health poor/fairly poor/intermediate vs. fairly good/good** |  |  | |  | |  | | 1.34*** | | 1.13 | | 1.59 | |  | | 1.34*** | 1.11 | | | 1.60 | | |  |  |
| **Physical activity no vs. yes** |  |  | |  | |  | | 1.09 | | 0.90 | | 1.31 | |  | | 1.05 | 0.87 | | | 1.28 | | |  |  |
| **Alcohol use no vs. yes** |  |  | |  | |  | | 0.71*** | | 0.57 | | 0.88 | |  | | 0.74** | 0.59 | | | 0.92 | | |  |  |
| **Common mental disorder no vs. yes** |  |  | |  | |  | | 0.83 | | 0.68 | | 1.02 | |  | | 0.86 | 0.69 | | | 1.05 | | |  |  |
| **Levels of engagement in cultural activity** |  |  | |  | |  | |  | |  | |  | |  | |  |  | | |  | | |  |  |
| high vs. low | 0.61*** | 0.50 | | 0.74 | |  | | 0.80** | | 0.65 | | 1.00 | |  | | 0.81** | 0.66 | | | 1.00 | | |  |  |
| intermediate vs. low | 0.74*** | 0.65 | | 0.85 | |  | | 0.91 | | 0.78 | | 1.06 | |  | | 0.92 | 0.78 | | | 1.08 | | |  |  |

19-year follow-up: Model 1=adjusted for age; Model 2=adjusted for age, area of residence, marital status, education, financial difficulties, obesity, chronic illness, smoking, alcohol consumption, self-rated health, common mental disorder and physical activity; Model 3=adjusted for age, area of residence, marital status, education, financial difficulties, obesity, chronic illness, smoking, alcohol consumption, self-rated health, common mental disorder and physical activity, censored for deaths occurring in first 3 years of follow-up. Note: *p<0.05, **p<0.01, ***p<0.001
